# Strengthening Teamwork and Respect (STAR): nurses’ perspectives and experiences of disrespectful maternity care in rural district hospitals in KwaZulu-Natal, South Africa

**DOI:** 10.1080/16549716.2026.2727792

**Published:** 2026-09-11

**Authors:** Christiane Horwood, Lyn Haskins, Silondile Luthuli, Sphindile Mapumulo, Veronique Filippi, Loveday Penn-Kekana, Tanya Doherty

**Affiliations:** a Centre for Research in Health Systems, University of KwaZulu-Natal, Durban, South Africa; b London School of Hygiene and Tropical Medicine, Keppel Street, London, UK; c Health Systems Research Unit, South African Medical Research Council, Cape Town, South Africa; d School of Public Health, University of the Western Cape, Cape Town, South Africa; e Department of Paediatrics and Child Health, University of Cape Town, Cape Town, South Africa

**Keywords:** Respectful maternity care, nurse midwives, attitudes of health personnel, quality of care, South Africa

## Abstract

**Background:**

Disrespectful maternal care has been reported from women’s perspectives, but less is known about how health workers understand these behaviours. To develop interventions, it is important to explore the drivers of disrespectful care from providers’ perspectives.

**Objective:**

To explore nurses’ perspectives on disrespectful maternal care.

**Methods:**

Semi-structured interviews were conducted with 18 nurses in maternity units in nine rural district hospitals in KwaZulu-Natal, South Africa, between April and August 2024. This was part of a continuous process evaluation for a participatory learning and action intervention called Strengthening Teamwork and Respect (STAR). One STAR champion and one team member were selected from each hospital. Interviews were recorded, transcribed, and thematically analysed using a reflexive thematic approach.

**Results:**

Participants reflected on how STAR changed their perceptions and understanding of respectful care. Five themes were identified: limited understanding of disrespectful care; normalisation of disrespectful behaviours; justification of disrespectful care; blaming women for disrespectful care; and perceptions of disrespect in the community. Overt incidents of disrespectful care were reported to be commonplace, including rudeness, shouting and slapping women. Lack of dignity and privacy was also common. Nurses recognised these behaviours as unacceptable and unprofessional and offered several explanations, including peer pressure, stress, burnout, and a poor work environment. Some believed that harsh behaviour was sometimes necessary to ensure safe delivery, particularly when women were perceived as uncooperative.

**Conclusions:**

Normalisation of disrespectful care highlights the need for interventions that address the underlying culture and the organisational and contextual factors that support and sustain these behaviours.

## Paper Context

- **Main findings:** Disrespectful care was firmly entrenched in the culture of maternity units and justified by nurses as being due to external factors and lack of cooperation from women.
- **Added knowledge:** A bottom-up participatory intervention that fostered critical reflection on the care provided was able to enrich nurses understanding of what was meant by disrespectful care and allowed them to question the underlying drivers of this behaviour.
- **Global health impact for policy and action:** Additional research is required to evaluate interventions to address the systemic and organisational culture issues that underpin disrespect and abuse in maternity units.

## Background

Improving the quality of facility-based care during labour and delivery is vital to improve outcomes for mothers and newborns, particularly in low-resource settings where high rates of maternal and perinatal mortality and morbidity remain a public health concern [1]. Interventions to improve maternal care have frequently focused on improving clinical practice and adherence to clinical guidelines, examples include training in emergency obstetric care (EmOC), and the WHO safe childbirth checklist [2,3]. However, in recent years, there has been increasing recognition of women’s experiences of care as a key component of quality maternal care [4].

Disrespectful care and abuse of women in labour has long been recognised as a global problem [5–7], leading to poor experiences of care that may discourage women from seeking facility-based care [8–10]. Disrespectful care during labour and delivery can be defined as comprising a number of elements: physical abuse, non-consented care, non-confidential care, non-dignified care, discrimination, abandonment of care, and detention in facilities [4,11,12]. The most vulnerable women, particularly younger women, those who are poor or have little formal education, are most at risk [13–15]. Several interventions implemented to improve respectful maternity care have had limited success, and there is much to learn about the most effective approaches [4,16,17]. Disrespectful care remains common in many settings [7,14], including South Africa [18,19].

Poor experiences of maternity care are associated with poor clinical outcomes for mothers and newborns. Evidence indicates that women with obstetric complications such as prolonged and obstructed labour, retained placenta, and excessive bleeding are more likely to experience disrespectful care [14,20,21]. Leite et al. further suggest that women who experience violence during childbirth are less likely to breastfeed exclusively [22]. In addition, abusive care can lead to prolonged psychological distress, with mothers experiencing depression, nightmares, panic attacks, and difficulty in forming a bond with their infant [23–26].

Therefore, a shift of focus is needed from a purely clinical model of care to a more integrated, person-centred approach, where women are actively involved in their own care [4]. WHO suggests that respectful maternity care is as important as clinical care and should be provided in a manner that maintains women’s dignity, privacy, and confidentiality; ensures freedom from harm; and allows informed choice and continuous support during childbirth [27]. Key components of respectful care include effective, culturally acceptable communication between health workers and women, the presence of a birth companion of the woman’s choice, and continuity of care from antenatal to postnatal care [28].

As a result, recent policy documents, including the revised 2024 South African maternity and perinatal care guidelines, have placed a stronger emphasis on the provision of respectful care [4,29]. In South Africa, routine delivery care is provided by trained midwives in public hospitals or midwife obstetric units, supported by generalist doctors on-site or at referral hospitals [30]. This study was conducted at the midpoint of STAR (Strengthening Teamwork and Respect), a bottom-up, participatory learning and action (PLA) intervention which aimed to improve care in rural maternity units. While many studies describe experiences of disrespectful care from women’s perspectives [7,19,31], to develop effective interventions, it is equally important to understand how health workers themselves perceive and experience these behaviours [8]. We present the findings of a qualitative study conducted in rural district hospitals in South Africa, which aimed to explore nurses’ perceptions and experiences of disrespectful maternal care and the social and organisational factors shaping this behaviour.

## Methods

This study was conducted as part of a mixed-methods evaluation of the STAR project. Using a qualitative research design, we conducted semi-structured interviews with nurses working in rural district hospital maternity units. Interviews explored health workers’ perceptions of maternal care in their hospital, with a particular focus on respectful care, and were part of a continuous process evaluation to document experiences of participating in STAR (30).

### Study setting

The study was conducted in maternity units in all nine hospitals situated in two districts of KwaZulu Natal (KZN), South Africa. Rural districts were purposively selected, in partnership with the KZN Department of Health, because of the challenges faced by hospitals in these areas, which frequently have limited staff and resources, and are situated far from specialist services. The districts, uMzinyathi and Zululand, are deep rural areas with poor basic infrastructure, sparse populations, and high unemployment, with 66% and 70% of the people living below the lower-level poverty line, respectively, and a high proportion of female-led households [32,33].

In South Africa, maternity services are provided free of charge, and coverage of facility-based delivery has been close to universal (>95%) for the past three decades [34]. District hospitals provide care for women in labour and the immediate postnatal period, including elective and emergency caesarean sections. Low risk vaginal deliveries are carried out by registered nurses, with midwifery training, assisted by enrolled (assistant) nurses. Some midwives complete an additional one-year training to become advanced diploma midwives and take a leadership role on the labour ward. While male nurses also have midwifery training, 88% of the nurses in KZN and most midwives working on labour wards are female [35]. Maternity units, which include the neonatal unit, are overseen by nurse managers.

While hospitals have all the equipment required to provide high-quality care during labour and delivery, most maternity units are overcrowded and understaffed. Labour wards usually comprise one room with several delivery beds, with curtains or screens available to pull around the beds.

### Description of the STAR intervention

The STAR intervention applied a participatory approach grounded in PLA theory to foster locally driven reflection, problem-solving, and accountability within the maternity units. The intervention involved training locally identified ‘champions’ to lead multidisciplinary STAR teams through a series of structured learning sessions guided by the STAR toolkit. The toolkit was developed by an intervention team of experts in adult education and organisational psychology, with input from provincial and district maternal health managers. The toolkit employed participatory activities, some of which were drawn from qualitative formative research with postpartum women and health professionals. Toolkit activities included exercises reflecting on team members’ motivations to become health workers, and a transect walk, ‘walking in the shoes of the patient’, to encourage reflection on women’s experiences of care.

Champions attended a two-day workshop on how to facilitate toolkit activities and were mentored by the intervention team through onsite visits, WhatsApp and phone call communication. STAR teams included doctors, midwives, enrolled nurses, quality managers, and maternity managers, and teams met regularly to work through toolkit activities. A mid-point workshop in each district brought champions together to share progress, and at the end of the intervention period, all nine facilities presented their change projects at a combined celebration workshop.

This study was conducted 6 months after the initiation of the STAR intervention. At the time of the interviews, STAR teams had been established and initial learning sessions conducted, focusing on reflecting on the maternity care provided, emphasising respectful care. However, STAR was at an early stage, and no change projects had been planned or implemented. The members of the intervention team were not involved in this analysis. Further details on the STAR intervention development are described elsewhere [36].

### Data collection

Semi-structured interviews were conducted with the champion or deputy champion and a STAR team member at each hospital to ensure diverse perspectives from a range of maternity staff, including nurse managers, nurses, midwives and assistant nurses, across all participating hospitals. Participants were purposively selected based on their availability and willingness to participate. Interviews explored participants’ perspectives of the care provided in the maternity unit and their experiences of respectful care.

We conducted 18 face-to-face interviews in nine hospitals between April and August 2024. Participants comprised nine STAR champions and nine STAR team members. All participants were nurses, including nurse managers, registered nurses, assistant nurses, and one nurse with an additional one-year of advanced midwifery training (Table 1). Interview duration ranged from 46 to 75 min. Participants were aged between 29 and 53 years.

**Table 1.** Characteristics of participants.

Participants	
Age	
<30 years	1
30–39	6
40–49	9
50–59	2
Gender	
Female	15
Male	3
Designation	
Advanced Diploma Midwife	1
Registered Nurse/Midwife	10
Assistant Nurse	1
Nursing Manager	6

Two of the investigators (SL and SM) conducted the in-depth interviews. Both are Zulu women with extensive experience in qualitative interviewing, trained to masters level. The researchers had no prior relationships with any of the interviewees and did not have a clinical background. All interviews were conducted in a private room at the health facility in English or the local language (IsiZulu), whichever the participants preferred. Only the participant and the interviewer were present during the interview.

### Analysis

Qualitative interviews were digitally recorded and transcribed verbatim. Transcripts were read and reread by members of the team (CH, LH, SL, SM) to familiarise themselves with the content and assess data saturation. The study was underpinned by an interpretivist qualitative methodological orientation, using reflexive thematic analysis to explore how nurses made sense of and understood respectful and disrespectful maternity care within their work context [37]. Initial inductive codes were generated from the data and discussed among the team to develop a preliminary coding framework. This framework was then applied to subsequent transcripts and refined iteratively as the analysis progressed.

Rigour and trustworthiness were enhanced through prolonged engagement with the data and investigator triangulation, with multiple researchers involved in the coding and interpretation. Regular team meetings were held to discuss coding and emerging themes. Reflexivity was maintained through ongoing discussions regarding how researchers’ backgrounds and perspectives may have influenced interpretation [38]. The analysis was managed using Nvivo v15 software.

## Results

We identified several interrelated themes that explained how disrespectful care was understood, justified and sustained in the maternity units. These were: limited understanding of respectful care; normalisation of disrespect and abuse through peer influence and workplace culture; nurses’ justifications for disrespectful care, including workplace pressures and structural constraints; blaming women for disrespectful care; and how disrespectful care affected the community.

### Limited understanding of respectful care

Overall, nurses demonstrated a narrow understanding of respectful care, with most equating it with the absence of overt abuse. All nurses were aware that it was considered inappropriate to be rude to mothers, shout at them, or hit them, but most lacked understanding and insight into the wider elements of respectful care, including dignity, confidentiality, privacy, joint decision-making, and consent. Several nurses stated that they had not heard of the term respectful care, ‘*It is a new term, totally new term. It’s just a new term. I have never heard of such’ (Hospital A, team member)*. Introduction of STAR had increased awareness of all aspects of disrespectful care.

I was aware of these things but I didn’t maybe look at them in-depth, so I can see that walking in bloodied clothes hurts the patient and they feel neglected. But when you read it, and you are hearing it from someone that went through that experience, as a person, when you do introspection, you realise that certain things that you thought were insignificant can affect a person’s self-esteem negatively, and they may feel reduced to nothing (Hospital F, champion, nurse manager)

We didn’t understand that it does not speak about respect per se. It is referring to the patient’s dignity and their privacy. Respect is an umbrella that has many things under it. That is when we understood that some of the things we are already doing, it’s just that we didn’t know that it relates to respectful care. A person will call me by any [bad] name and I will laugh [chuckles] but continue to assist them. So, yes, it is a sign of respect for you to introduce yourself to the patient. Telling the patient what treatment you are going to give them is also showing respectful care, because you are involving them. (Hospital E, deputy champion, registered nurse)

However, a few participants noted the increased emphasis on respectful care in the newly released South African guidelines for maternal and perinatal care, where the first chapter is about respectful care [29]. This provided strong motivation and support for maternity staff to prioritise respectful care.

Looking at our new perinatal and maternity guidelines, the 2024 [guidelines], that were just introduced. Just when you start, in the first page, they are talking about respectful care. So, I was even telling the team that “guys, can you see that respectful care is a must because even our guidelines are now talking about respectful care”. (Hospital G, champion, nurse manager)

### Normalisation of disrespect and abuse through peer influences and workplace culture

Incidents of disrespectful care were widely reported by nurses in all hospitals, suggesting that such practices were part of the organizational culture. Participants also spoke of such incidents while describing their work environment more generally, sometimes without recognising that the behaviour fell within the definition of disrespectful care. Incidents most often referred to were rudeness, shouting at and hitting women. Other behaviours alluded to by participants included inadequate privacy, lack of dignity, failure to communicate with mothers about their care, and failure to obtain consent for procedures.

With respect for patients, we are nowhere. Hence, I said in sick antenatal [ward], they were having the same gap to say staff is rude, they are rude. In labour ward, I am working there, they will even beat the patient if the patient does not want to push because of her [own] reasons [smacking sound]. So, it’s like, even here [in postnatal ward], if a woman is not breastfeeding, oh, you will see, “why are you not breastfeeding? You should not have given birth if you did not want to [breastfeed].” So, those negative and disrespectful statements come from nurses. (Hospital A, team member, registered nurse)

Most participants were aware that their behaviour was contrary to professional standards and highlighted that being rude and dismissive to women went against their core mission as nurses and caregivers, suggesting that disrespectful behaviour was not only due to lack of awareness but was sustained by the context in which care was delivered. Participants also reported that by emphasising respectful care, STAR had reminded them of the core values that underpin the nursing profession ‘*It is taking us back to what nursing is about’ (Hospital D, champion, registered nurse*), prompting reflection on care practices and challenging previously accepted behaviours.

So, it revives those things of seeing a patient as she is, in a vulnerable state where pain is what is on her mind and making her do things wrongly sometimes. So, you must understand and speak to the woman in a manner that the woman does not feel embarrassed or degraded, but in a way that she will still feel as an important person or as a human being when you interact with her. Even if you are correcting her, the way you speak, you still preserve that she is a human being. (Hospital E, team member, nurse manager)

Several participants highlighted that abusive and disrespectful care was reinforced by peer pressure and professional socialisation, which encouraged them to adopt the same behaviour as their colleagues, and discouraged them from challenging such practices. Several reported that when they started working in maternity, they were surprised to see the way women were treated, but they gradually adopted similar behaviours, suggesting that informal social norms were prioritised over adherence to professional standards.

I think this thing [treating patient with respect] got lost, like, I will say through peer pressure, to make it understandable. Peer pressure. When I arrived [in maternity], and find that it is being done, I will also do it. Do you see that? As much as maybe I knew what the right thing to do is, but because this is how everyone works, I will also do it, you see (Hospital D, champion, registered nurse)

Further, nurses highlighted that although the staff themselves were aware that their behaviour was disrespectful, they were fearful of challenging their colleagues when they observed or participated in disrespectful care, suggesting that fear of conflict contributed to sustaining disrespectful behaviour.

I learned that they [maternity staff] are aware that we don’t treat our patients with the respect that they deserve. They are aware of it but they are afraid to voice it out. I learned that some of them are culprits of the disrespect and destroying team work (Hospital A, champion, advanced midwife)

In addition, the same participant described how disrespectful care could easily occur in their own practice, due to high patient volumes and repetitive work, making it is easy to forget that each woman is an individual who needs to be treated with respect.

When you have done the same job for a long time it ends up feeling like you are used to it, whereas the patient is not used to it. You see a different person every day. So, when you see a patient come in, you just jump into business without introducing yourself or asking them how they feel. You treat them as if you are continuing to provide care to someone that you had seen yesterday or previously. (Hospital A, champion, advanced midwife)

Some participants explained that colleagues on the ward actively endorsed harsh behaviour towards women, saying that it was appropriate in some situations, and several expressed their own support for this view. Nurses reported that it was sometimes considered ‘necessary’ to shout at women who were not obeying instructions, suggesting that disrespectful practices were legitimised as appropriate or necessary and in this way became further entrenched.

There in the labour ward, there are, you know in the labour ward sometimes with the patients, you must raise your voice a little bit. (Hospital I, champion, nurse manager)

So, you find that a person [colleague] just doesn’t want to change, you see. No matter how hard you try. But we will keep on trying. We will not stop. But there are those people that you know that it is like pouring water on the back of a duck. (Hospital F, champion, nurse manager)

One participant reported calling out other health workers when she observed disrespectful behaviour, and how this negatively affected her relationship with colleagues, highlighting the risks associated with challenging dominant norms. She further highlighted that staff needed assistance from managers to address disrespectful care.

They [colleagues] will hate you, and with me, they did hate me but they are now fine with me because I stood by what I was telling them to say, “you are not going to beat the patient in front of me, otherwise I will report you.” And one of them said, she is present today, she said, “He is judging us because he just came [to work in maternity]. He does not know these patients are rude.” And the managers, they need to be there as well because we can’t always report, “so and so has beaten the patient”, because they will say you are a snitch. So, the presence of the managers, they see the true picture of how we approach the patient. (Hospital A, team member, registered nurse)

### Justifications for disrespectful care

Nurses suggested a variety of reasons for the high number of incidents of disrespectful care, including the work environment, short staffing, and patients’ behaviour.

### Workplace pressures and structural constraints

Nurses frequently attributed disrespectful care to structural constraints, including a stressful work environment, poor infrastructure, overcrowding and lack of resources, making it difficult to maintain privacy and dignity and provide respectful care. Participants’ accounts suggest that structural factors were not only barriers to respectful care but also shaped how disrespectful practices were understood and justified in maternity units.

So, for improvement with respectful care, [it is] just that our structures sometimes do not allow us. For an example, our delivery room, we are having three beds there, but it’s supposed to have two, when you see the screens … we need to squeeze another bed because we’ve got a lot of patients. So, you will see sometimes that our screening is not that adequate (Hospital G, champion, nurse manager)

A major challenge that many participants linked to disrespectful care was the shortage of staff and resulting work pressure, described as sources of stress for nurses. At times, staff members were unable to take their scheduled meal breaks if the ward was busy, leading to fatigue and frustration. As one nurse explained, it is difficult to be polite when you are hungry. This suggests that when disrespectful interactions emerge in the context of fatigue and competing demands, respectful care is perceived as secondary to clinical care.

Because of the circumstances that we work under, as I am saying, that here you can get so busy that you can’t even eat. You know the saying that hunger causes anger? … . So, I am hungry. Do you think I would have time to talk to them politely? (Hospital F, champion, nurse manager)

However, not all participants accepted these explanations, and some participants explicitly challenged the idea that structural constraints could justify disrespectful and abusive behaviour, indicating that individual attitudes also play a role in shaping care practices.

The mere fact that you are thinking that the reason why they [nurses] are beating [women] is because of the shortage [of staff]. So, I won’t say no, but I will say, to add, I think the reason why they are beating patients is because they are rude … shortage doesn’t mean you need to beat, you see, because the off duties [days off] will give you time to go and rest. (Hospital A, team member, registered nurse)

### Attribution of blame to women

Nurses often attributed their disrespectful behaviour to the women’s conduct, describing their harsh behaviour as a reaction to women perceived as ‘uncooperative’ or ‘difficult’ and that their harsh behaviour was only directed towards these women, thereby framing disrespectful care as conditional on womens’ compliance, rather than the responsibility of the health worker.

Sometimes it becomes more difficult to, ok, to give respectful care because sometimes you are faced with a difficult client, you see. You want to help the client, but she just becomes difficult and then to give respectful care, sometimes it’s not easy, but we are trying to get there. (Hospital C, deputy champion, registered nurse)

In addition, nurses expressed that responding with equal rudeness to a patient who was rude was viewed as acceptable and were surprised that such behaviour might be considered inappropriate and unprofessional. This suggests that norms around respectful care were inconsistently understood, and that respectful care was perceived as unrealistic or unnecessary in situations where patients were seen as intentionally non-compliant.

It was a challenge at the beginning when we first started and didn’t understand what the concept of respectful care is. Which means that we must give them [patients] respect even if they are rude to us? No matter how a person addresses you, you must just keep smiling? Someone said that. So, we must just keep smiling even when you see that a person is doing something wrong intentionally? (Hospital E, deputy champion, registered nurse)

In addition, several nurses linked disrespectful care to prevention of adverse outcomes for mothers and babies, framing harsh behaviour as necessary to achieve a good outcome. Nurses described being fearful of being blamed for an adverse outcome, and feeling compelled to shout or scold women who were perceived as putting themselves or their babies at risk. In this way, disrespectful practices were justified as a form of risk management and rationalised as being in the best interests of the mother and baby.

I will cross the line. I will remember afterwards that I messed up … because it happens that a person really doesn’t want to open their legs, and you can see the baby’s head, and the baby might die and you will get in trouble. But when you have finished, go back to her and tell her that you were not fighting, you just wanted her to deliver her child safely (Hospital F, champion, nurse manager)

In contrast, some nurses emphasised that the responsibility to provide respectful care rests with health workers, regardless of women’s behaviour, framing respectful care as a core professional obligation requiring them to remain calm and compassionate in all circumstances. This suggests that professional norms were not perceived consistently among maternity staff.

When the patients they come in labour they are still human beings. Yes, they will do things that will push you or rub you the wrong way, but try to understand the patients and, also, we are reminding ourselves that we are there for them. They depend on us. So, we must care for them. We must watch how we talk. (Hospital E, team member, nurse manager)

### Consequences of disrespectful care

Litigation was frequently referred to, with many nurses expressing fear of complaints being made against them. Nurses explicitly linked disrespectful care to the increasing number of complaints and litigation in their hospitals and recognised that providing respectful care could improve women’s birth experiences, thereby reducing complaints and litigation. Several participants highlighted the role of communication in shaping women’s experiences of care, noting that clear explanations helped women understand what was happening and reduced the likelihood of women feeling mistreated or making a complaint. Thus, communication was seen, not only as a component of respectful care but also as protective against conflict and formal complaints

There are things that you do, that we used to do without paying much attention about how important it is to first communicate with the patient, which ended up causing problems for us because we didn’t explain to the patient. But after STAR we can now communicate with the patient and explain what you will do, why are you doing it and so on. That helps in prevention and reduction of the number of litigations. Because really with some people, if you don’t communicate or explain to them properly, they might make their own assumptions, whereas that is not what you meant (Hospital I, team member, registered nurse)

In addition, participants noted that the wider community was aware of incidents of abuse and disrespect in the maternity unit, which contributed to its reputation in the community as a hostile and unsafe environment. Women were said to arrive on the ward feeling anxious and expecting mistreatment, particularly young women or teenagers without previous childbirth experience. Nurses acknowledged that this could influence women’s behaviour, with anxiety and distress sometimes being interpreted by staff as a poor attitude or a lack of cooperation.

People [in the community] they talk about, especially when you are a teenager, “you will meet the midwives there, they are going to hit you” you see, all those things. So, in any case the challenges, they are being afraid of us because they’ve been told about whatever. (Hospital G, champion, nurse manager)

## Discussion

Respectful, high-quality care during labour and delivery is a fundamental right for all women [28], and experiences of disrespectful and abusive care may lead to lasting clinical and psychological harm [8,20]. These findings demonstrate that nurses regarded overt incidents of disrespectful care as commonplace and normalised, while also acknowledging such behaviour as unacceptable and unprofessional. Participants described how the STAR project to improve teamwork and respectful care, prompted maternity teams to reflect on the care provided, raised awareness and led nurses to new understandings of respectful care. Nurses, some of whom had never heard the term respectful care, were reminded that respect and empathy are core nursing values and reported that STAR enabled them to see respectful care as more than the absence of physical abuse, prompting them to consider underlying drivers of disrespect and abuse.

Developing effective strategies to address disrespectful maternal care requires a clear understanding of the underlying complexities and drivers of this behaviour from the health workers perspective. Disrespectful care arises from a complex interaction between health workers’ attitudes and the wider context in which they live and work, including the policy framework, structural constraints and work environment, as well as the wider cultural and social environment [6]. In our setting, even severe elements of disrespect and abuse were normalised, and this appeared to be specific to the maternity unit. Nurses expressed surprise at seeing disrespectful behaviour on starting work in maternity, but quickly became socialized into the culture of disrespect, reinforced by peer pressure and fear of conflict with colleagues.

Nurses clearly acknowledged that rudeness, shouting and slapping women during labour and delivery were unacceptable, unprofessional and contrary to nursing values. Similar to other studies, they provided several justifications for this behaviour, blaming peer pressure, understaffing, lack of resources, poor infrastructure and difficult work conditions [39]. They also frequently blamed women’s apparently uncooperative behaviour, expressing limited empathy for women who were vulnerable and in pain. Notably, participants mainly described their colleagues’ disrespectful behaviour rather than acknowledging their own, suggesting that recognising their own contribution to disrespectful care may be challenging, and highlights the importance of critical self-reflection to achieve behaviour change.

Normalisation of disrespect and abuse has been widely reported, primarily from the women’s perspective, with many women reporting that they accept such practices as routine while also reporting feelings of helplessness and dehumanisation as a result [8,40]. Previous research has proposed several explanations, including that inherent hierarchy in the health system fosters top-down behaviours, so that health workers receiving harsh treatment from their seniors repeat this behaviour towards their patients [12,41,42]. Other researchers suggest that disrespectful behaviour reflects health workers’ attempts to assert control and maintain power, as they perceive themselves to have a different status from the women in their care [6,10,43,44]. Jewkes et al. argued that harsh behaviour may serve as a way for nurses to assert their status and underpin the perceived inferior status of the women [6]. This view is supported by evidence that women of lower education and socioeconomic status are more likely to experience disrespect. A further explanation links disrespectful care to the broader social position of women, so that abusive behaviour is a means to exert power by health workers who feel disempowered in their work or social environments [45].

Another factor contributing to the normalisation of disrespectful and abusive care was the perception that shouting and slapping women, particularly during delivery, improved clinical outcomes. Several nurses agreed with this, particularly for women considered difficult or uncooperative, and described fear of being blamed for an adverse obstetric outcome. Participants did not express empathy or acknowledge that their perception of women’s uncooperative behaviour may reflect a lack of understanding of the diversity of delivery trajectories. Similar findings have been reported in several African studies [10,43,46,47], including a recent Tanzanian study, where health workers associated abuse with positive birth outcomes and reported that women supported the practice as being for their own good [41]. However, this behaviour may also reflect attempts to exert control or punish women for what was perceived as their poor behaviour [44].

While health workers in our study were aware of some aspects of disrespectful care, they lacked awareness of others, such as maintaining privacy and dignity and obtaining consent for examinations and procedures. This is supported by the findings of a recent survey among women who delivered in the same rural hospitals, which reported lack of privacy, unconsented care and poor communication with health workers [48]. While still at the early stage of implementation, STAR had raised awareness of these issues, as shown by one participant who expressed surprise that asking women to walk around in a blood-stained gown was disrespectful and violated her right to dignity. Interventions to address disrespectful care must ensure that health workers understand all elements of respectful care, as set out in the new SA Maternity guidelines [29]. While more research is required to evaluate effective interventions to address disrespectful care, several studies have shown that health worker training, either alone or combined in a multi-component intervention, may improve the quality of maternal care [16]. However, given the complex nature of disrespect and abuse, multi-component interventions that include skills development in counselling and communication, are more likely to be effective [49].

Our findings suggest that interventions must address the underlying organisational culture in maternity units, which is reinforced by health workers themselves. Health workers operate in a complex environment where they must navigate competing pressures from professional responsibility, fear of blame, and a deep-rooted institutional culture. The belief that disrespectful behaviour may prevent adverse outcomes, with health workers fearing being held personally accountable, suggests a culture of blame within maternity units [50]. This fear shapes their interactions with women and may contribute to disrespect and abuse. Addressing these behaviours requires interventions that not only promote respectful care at an individual level but also reform the underlying system and culture that perpetuates these practices. We suggest that a bottom-up participatory approach, as used by STAR, can enable health workers to critically reflect on care practices, identify changes and take ownership of solutions to improve quality of care. In addition, as highlighted by participants, management engagement is crucial for supporting behaviour change, particularly ongoing supportive supervision and skills development. While many drivers of disrespectful behaviour are structural and cannot be easily addressed, such as understaffing, poor infrastructure and lack of resources, the recent WHO Compendium on respectful maternal and newborn care suggests approaches to ‘Care for Carer’s’ within under-resourced health systems, including respect for health workers’ rights, peer support and counselling, mentorship, supportive supervision, and appointment of champions to serve as role models [51].

Any intervention to comprehensively address disrespectful care should include a community component [51]. Participants noted that stories of abusive care in the community may influence women’s behaviour, so that women perceived as uncooperative may just be anxious and fearful of abuse. This suggests a self-perpetuating cycle of anxiety, abusive behaviour and poor experiences of care, which may contribute to the high incidence of litigation in maternity units. This is supported by a recent study among women who recently delivered in the same hospitals, which found that women adopted defensive behaviours to avoid conflict with health workers [48]. Community interventions should ensure that women know their rights and are empowered to constructively engage with health workers to request information about their care, provide consent for procedures, and request pain relief when required. Education could be provided during antenatal visits or through community workshops and maternity open days to address women’s expectations of labour and delivery [16,51,52]. As part of multi-component interventions, patients’ rights charters have been successfully implemented to improve quality of care [53]. Access to a companion of the woman’s choice is a pillar of quality of care; while this has been supported by policy since 2016 [54], new maternity guidelines provide stronger support for birth companions [29]. Birth companionship has been shown to be protective against disrespect and abuse and improves women’s experiences of labour [14,31,55]. However, the implementation of birth companionship has been challenging in South Africa because of inadequate space and privacy, poor infrastructure and staff shortages [56].

### Strengths and limitations

This study employed a robust qualitative approach to explore this topic using a team of experienced researchers. However, interviews were conducted in the workplace, albeit in a private area, and the sensitive nature of the topic may have limited participants’ willingness to fully describe the abusive behaviour of themselves or their colleagues, leading to social desirability bias. All participants were nurses or midwives, and the perspectives of doctors who are important leaders and role models in maternity units were not included.

## Conclusion

This study highlights the complex interplay of issues underpinning disrespectful maternal care from the perspectives of nurses who observed and engaged in this behaviour. Abusive behaviours were found to be commonplace, normalised and deeply entrenched in the organisational culture of maternity units. These findings suggest that, to be effective, interventions should address disrespectful behaviour, not only at the individual level but also at the team, facility and community levels. Providing health workers with the space to reflect, explore the reasons for poor care, and facilitating them to identify their own solutions can be a powerful tool for change [36]. Improvements to the work environment would further support changes in practice. Further research is required to build a stronger evidence base for interventions to improve respectful care.

## Supplementary Material

COREQ_respectfulcareGHA.pdf

## Data Availability

The data supporting the findings of the current study cannot be made publicly available because of the sensitive nature of the data, which could allow participants and health facilities to be identified.

## References

[1] Boerma T, Requejo J, Victora CG, et al. Countdown to 2030: tracking progress towards universal coverage for reproductive, maternal, newborn, and child health. Lancet. 2018;391:1538–12. doi: 10.1016/S0140-6736(18)30104-1 29395268

[2] World Health Organization. Monitoring emergency obstetric care: a handbook. Geneva: World Health Organization; 2009.

[3] World Health Organization. WHO safe childbirth checklist implementation guide: improving the quality of facility-based delivery for mothers and newborns. Geneva: World Health Organization; 2015. p. 9241549459.

[4] World Health Organization. Compendium on respectful maternal and newborn care. Geneva: World Health Organization; 2025. Licence: CC BY-NC-SA 3.0 IGO.

[5] Rosen HE, Lynam PF, Carr C, et al. Direct observation of respectful maternity care in five countries: a cross-sectional study of health facilities in East and Southern Africa. BMC Pregnancy Childbirth. 2015;15:306. doi: 10.1186/s12884-015-0728-4 PubMed PMID: 26596353; PubMed Central PMCID: PMC4657214. 26596353 PMC4657214

[6] Jewkes R, Abrahams N, Mvo Z. Why do nurses abuse patients? Reflections from South African obstetric services. Soc Sci Med (1982). 1998;47:1781–1795. doi: 10.1016/S0277-9536(98)00240-8 PubMed PMID: 9877348. 9877348

[7] Kassa ZY, Tsegaye B, Abeje A. Disrespect and abuse of women during the process of childbirth at health facilities in sub-Saharan Africa: a systematic review and meta-analysis. BMC Int Health Hum Rights. 2020;20:23. doi: 10.1186/s12914-020-00242-y 32894127 PMC7487593

[8] Amathullah AS, Rishard M, Walpita Y. Impacts of disrespectful care and abusive care practices in maternity units and potential interventions to improve the quality of care in low- and middle-income countries: a narrative review. Int J Gynaecol Obstet. 2023;162:847–859. Epub 20230428. doi: 10.1002/ijgo.14811 PubMed PMID: 37118934. 37118934

[9] Bohren MA, Hunter EC, Munthe-Kaas HM, et al. Facilitators and barriers to facility-based delivery in low-and middle-income countries: a qualitative evidence synthesis. Reprod Health. 2014;11:71. Available from: 1742-4755-11-71.pdf 25238684 10.1186/1742-4755-11-71 PMC4247708

[10] Mannava P, Durrant K, Fisher J, et al. Attitudes and behaviours of maternal health care providers in interactions with clients: a systematic review. Global Health. 2015;11:36. Available from: s12992-015-0117-9.pdf 26276053 10.1186/s12992-015-0117-9 PMC4537564

[11] Bowser D, Hill K. Exploring evidence for disrespect and abuse in facility-based childbirth: report of a landscape analysis. USAID-Traction project; 2010. Available from: https://coregroup.org/wp-content/uploads/media-backup/documents/elluminates/Respectful_Care_at_Birth_9-20-101_Final.pdf

[12] Bohren MA, Vogel JP, Hunter EC, et al. The mistreatment of women during childbirth in health facilities globally: a mixed-methods systematic review. PLoS Med. 2015;12:e1001847; discussion e. Epub 2015/07/01. doi: 10.1371/journal.pmed.1001847 PubMed PMID: 26126110; PubMed Central PMCID: PMC4488322. PMC4488322 26126110

[13] Oosthuizen SJ, Bergh A-M, Pattinson RC, et al. It does matter where you come from: mothers’ experiences of childbirth in midwife obstetric units, Tshwane, South Africa. Reprod Health. 2017;14:151. Available from: s12978-017-0411-5.pdf 29145897 10.1186/s12978-017-0411-5 PMC5689145

[14] Maldie M, Egata G, Chanie MG, et al. Magnitude and associated factors of disrespect and abusive care among laboring mothers at public health facilities in Borena District, South Wollo, Ethiopia. PLoS One. 2021;16:e0256951. Available from: journal.pone.0256951(4).pdf 34793460 10.1371/journal.pone.0256951 PMC8601571

[15] Afulani PA, Okiring J, Aborigo RA, et al. Provider implicit and explicit bias in person-centered maternity care: a cross-sectional study with maternity providers in Northern Ghana. BMC Health Serv Res. 2023;23:254. Available from: s12913-023-09261-6.pdf 36918860 10.1186/s12913-023-09261-6 PMC10015736

[16] Mira-Catala P, Hernandez-Aguado I, Chilet-Rosell E. Respectful maternity care interventions to address women mistreatment in childbirth: what has been done? BMC Pregnancy Childbirth. 2024;24:322. Available from: s12884-024-06524-w.pdf 38671343 10.1186/s12884-024-06524-w PMC11046783

[17] Amathullah AS, Rishard M, Walpita Y. Impacts of disrespectful care and abusive care practices in maternity units and potential interventions to improve the quality of care in low-and middle-income countries: a narrative review. Int J Gynecol Obstet. 2023;162:847–859. 10.1002/ijgo.14811 37118934

[18] Doherty T, Horwood C, Mapumulo S, et al. Case for improving respectful care: results from a cross-sectional survey of person-centred maternity care in rural South Africa. BMJ Public Health. 2024;2:1–12. Available from: e001086.full.pdf 10.1136/bmjph-2024-001086 PMC11816963 40018554

[19] Mapumulo S, Haskins L, Luthuli S, et al. Health workers’ disrespectful and abusive behaviour towards women during labour and delivery: a qualitative study in Durban, South Africa. PLoS One. 2021;16:e0261204. Epub 20211214. PubMed PMID: 34905562; PubMed Central PMCID: PMC8670673. journal.pone.0261204.pdf 34905562 10.1371/journal.pone.0261204 PMC8670673

[20] Raj A, Dey A, Boyce S, et al. Associations between mistreatment by a provider during childbirth and maternal health complications in Uttar Pradesh, India. Matern Child Health J. 2017;21:1821–1833. doi: 10.1007/s10995-017-2298-8 28676965

[21] Yohannes E, Moti G, Gelan G, et al. Impact of disrespectful maternity care on childbirth complications: a multicentre cross-sectional study in Ethiopia. BMC Pregnancy Childbirth. 2024;24:380. Available from: s12884-024-06574-0.pdf 38773395 10.1186/s12884-024-06574-0 PMC11110437

[22] Leite TH, Marques ES, Mesenburg MA, et al. The effect of obstetric violence during childbirth on breastfeeding: findings from a perinatal cohort “Birth in Brazil”. Lancet Reg Health. 2023;19:100438. doi: 10.1016/j.lana.2023.100438 PMC9975306 36874165

[23] Silveira MF, Mesenburg MA, Bertoldi AD, et al. The association between disrespect and abuse of women during childbirth and postpartum depression: findings from the 2015 Pelotas birth cohort study. J Affect Disord. 2019;256:441–447. doi: 10.1016/j.jad.2019.06.016 31252237 PMC6880287

[24] Adewuya A, Ologun Y, Ibigbami O. Post-traumatic stress disorder after childbirth in Nigerian women: prevalence and risk factors. BJOG: Int J Obstet Gynaecol. 2006;113:284–288. doi: 10.1111/j.1471-0528.2006.00861.x 16487199

[25] Guure C, Aviisah PA, Adu-Bonsaffoh K, et al. Mistreatment of women during childbirth and postpartum depression: secondary analysis of WHO community survey across four countries. BMJ Glob Health. 2023;8:1–11. Available from: e011705.full.pdf 10.1136/bmjgh-2023-011705 PMC10450127 37612033

[26] Paiz JC, de Jezus Castro SM, Giugliani ERJ, et al. Association between mistreatment of women during childbirth and symptoms suggestive of postpartum depression. BMC Pregnancy Childbirth. 2022;22:664. Available from: s12884-022-04978-4.pdf 36028806 10.1186/s12884-022-04978-4 PMC9413948

[27] Tunçalp Ӧ, Were W, MacLennan C, et al. Quality of care for pregnant women and newborns—the WHO vision. BJOG. 2015;122:1045. Available from: BJO-122-1045.pdf 25929823 10.1111/1471-0528.13451 PMC5029576

[28] World Health Organization. WHO recommendations on intrapartum care for a positive childbirth experience. Geneva: World Health Organization; 2018. 30070803

[29] South Africa National Department of Health. National integrated maternal and perinatal care guidelines for South Africa. 5th ed. In: National Department of Health, editor. Pretoria: National Department of Health; 2024. p. 16–20.

[30] SA National Department of Health, Statistics South Africa, SA Medical Research Council. South Africa demographic and health survey 2016: key indicators. Pretoria, South Africa: Department of Health; 2017.

[31] Mirzania M, Shakibazadeh E, Maleki A, et al. Prevalence of mistreatment and disrespect of women during childbirth in the world: a systematic review and meta-analysis. Reprod Health. 2025;22:193. Available from: s12978-025-02119-6.pdf 41088180 10.1186/s12978-025-02119-6 PMC12522719

[32] Department of Cooperative Governance and Traditional Affairs. Profile and analysis Umzinyathi district municipality, KZN. Pretoria, South Africa: Cooperative governance and traditional affairs; 2020.

[33] Department of Cooperative Governance and Traditional Affairs. Profile and analysis Zululand district municipality, KZN. Pretoria, South Africa: Cooperative governance and traditional affairs; 2020.

[34] South African National Department of Health SSA. SA Medical Research Council. 2016 South African demographic and health survey. Pretoria: SA National Department of Health; 2016. Available from: FR337.pdf

[35] South African Nursing Council. Provincial distribution of nursing manpower versus the population of the Republic of South Africa as at 31 December 2022. 2023. Available from: Stats-2022-1-Provincial-Distribution.pdf

[36] Doherty T, Petrus R, Land S, et al. Strengthening teamwork and respect (STAR) in maternity units: developing a health system intervention in South Africa. Glob Health Action. 2025;18:2440982. doi: 10.1080/16549716.2024.2440982 39898401 PMC11792138

[37] Braun V, Clarke V. Using thematic analysis in psychology. Qualitative Res Phychol. 2006;3:77–101. doi: 10.1191/1478088706qp063oa

[38] Braun V, Clarke V. Reflecting on reflexive thematic analysis. Qual Res Sport Exer Health. 2019;11:589–597. doi: 10.1080/2159676X.2019.1628806

[39] Buback L, Kinyua J, Akinyi B, et al. Provider perceptions of lack of supportive care during childbirth: a mixed methods study in Kenya. Health Care Women Int. 2022;43:1062–1083. doi: 10.1080/07399332.2021.1961776 34534038 PMC9080303

[40] Orpin J, Puthussery S, Davidson R, et al. Women’s experiences of disrespect and abuse in maternity care facilities in Benue State, Nigeria. BMC Pregnancy Childbirth. 2018;18:213. Available from: s12884-018-1847-5.pdf 29879944 10.1186/s12884-018-1847-5 PMC5992700

[41] Mwasha LK, Kisaka LM, Pallangyo ES. Disrespect and abuse in maternity care in a low-resource setting in Tanzania: Provider’s perspectives of practice. PLoS One. 2023;18:e0281349. Epub 20230322. PubMed PMID: 36947537; PubMed Central PMCID: PMC10032509. journal.pone.0281349.pdf 36947537 10.1371/journal.pone.0281349 PMC10032509

[42] Fonn S, Xaba M. Health workers for change: developing the initiative. Health Policy Plan. 2001;16:13–18. Available from: 160013.pdf 10.1093/heapol/16.suppl_1.13 11599664

[43] Mehretie Adinew Y, Kelly J, Marshall A, et al. Care providers’ perspectives on disrespect and abuse of women during facility-based childbirth in Ethiopia: a qualitative study. Int J Women’s Health. 2021;Volume 13:1181–1195. Available from: https://www.tandfonline.com/doi/pdf/10.2147/IJWH.S333863 10.2147/IJWH.S333863 PMC8643202 34876861

[44] Bradley S, McCourt C, Rayment J, et al. Midwives’ perspectives on (dis) respectful intrapartum care during facility-based delivery in sub-Saharan Africa: a qualitative systematic review and meta-synthesis. Reprod Health. 2019;16:116. Available from: s12978-019-0773-y.pdf 31345239 10.1186/s12978-019-0773-y PMC6659209

[45] Schaaf M, Jaffe M, Tunçalp Ö, et al. A critical interpretive synthesis of power and mistreatment of women in maternity care. PLoS Global Public Health. 2023;3:e0000616. Available from: journal.pgph.0000616.pdf 36962936 10.1371/journal.pgph.0000616 PMC10021192

[46] Okedo-Alex IN, Akamike IC, Nwafor JI, et al. Multi-stakeholder perspectives on the maternal, provider, institutional, community, and policy drivers of disrespectful maternity care in South-East Nigeria. Int J Women Health. 2020;12:1145–1159. doi: 10.2147/IJWH.S277827 PMC7733334 33324116

[47] Wesson J, Hamunime N, Viadro C, et al. Provider and client perspectives on maternity care in Namibia: results from two cross-sectional studies. BMC Pregnancy Childbirth. 2018;18:363. Epub 20180905. doi: 10.1186/s12884-018-1999-3 PubMed PMID: 30185161; PubMed Central PMCID: PMC6126026. 30185161 PMC6126026

[48] Luthuli S, Horwood C, Filippi V, et al. Women’s experiences of communication and supportive care during labour: a qualitative study in rural KwaZulu-Natal, South Africa. BMC Pregnancy Childbirth. 2025;25:1157. Epub 20251103. doi: 10.1186/s12884-025-08334-0 PubMed PMID: 41184771; PubMed Central PMCID: PMC12581260. 41184771 PMC12581260

[49] Kasaye H, Sheehy A, Scarf V, et al. The roles of multi-component interventions in reducing mistreatment of women and enhancing respectful maternity care: a systematic review. BMC Pregnancy Childbirth. 2023;23:305. Available from: s12884-023-05640-3.pdf 37127582 10.1186/s12884-023-05640-3 PMC10150509

[50] Horwood C, Filippi V, Haskins L, et al. Reflection or correction? A qualitative study of health manager’s experience of clinical audit activities in rural maternity units in KwaZulu-Natal, South Africa. Glob Public Health. 2025;20:2597114. Epub 20251204. doi: 10.1080/17441692.2025.2597114 PubMed PMID: 41342233. 41342233

[51] World Health Organization. Compendium on respectful maternal and newborn care. Geneva: World Health Organization; 2025.

[52] Downe S, Lawrie TA, Finlayson K, et al. Effectiveness of respectful care policies for women using routine intrapartum services: a systematic review. Reprod Health. 2018;15:23. Available from: s12978-018-0466-y.pdf 29409519 10.1186/s12978-018-0466-y PMC5801845

[53] Kujawski SA, Freedman LP, Ramsey K, et al. Community and health system intervention to reduce disrespect and abuse during childbirth in Tanga region, Tanzania: a comparative before-and-after study. PLoS Med. 2017;14:e1002341. Available from: file.pdf 28700587 10.1371/journal.pmed.1002341 PMC5507413

[54] South Africa National Department of Health. Guidelines for maternity care in South Africa: a manual for clinics, community health centres and district hospitals. Pretoria: National Department of Health Pretoria; 2015.

[55] Kasaye H, Scarf V, Sheehy A, et al. The mistreatment of women during maternity care and its association with the maternal continuum of care in health facilities. BMC Pregnancy Childbirth. 2024;24:129. Available from: s12884-024-06310-8.pdf 38350892 10.1186/s12884-024-06310-8 PMC10863180

[56] Summerton JV, Mtileni TR, Moshabela ME. Experiences and perceptions of birth companions supporting women in labour at a district Hospital in Limpopo, South Africa. Curationis. 2021;44:e1–e7. Epub 20211027. PubMed PMID: 34797104; PubMed Central PMCID: PMC8603091. summerton-et-al-2021-experiences-and-perceptions-of-birth-companions-supporting-women-in-labour-at-a-district-hospital.pdf 10.4102/curationis.v44i1.2186 PMC8603091 34797104

